# Support services for informal caregivers of community-dwelling frail elderly: a systematic review

**Published:** 2012-09-04

**Authors:** Maja Lopez Hartmann, Johan Wens, Veronique Verhoeven, Roy Remmen

**Affiliations:** Department of Primary and Interdisciplinary Care, University of Antwerp, Belgium; Department of Primary and Interdisciplinary Care, University of Antwerp, Belgium; Department of Primary and Interdisciplinary Care, University of Antwerp, Belgium; Department of Primary and Interdisciplinary Care, University of Antwerp, Belgium

**Keywords:** frail elderly, caregivers, health services needs and demand

## Abstract

**Purpose:**

The aim of this study is to review the current best evidence on the effectiveness of different types of support services targeting informal caregivers of community-dwelling frail elderly.

**Theory:**

Informal caregivers are important resources for community-dwelling frail elderly. But caring can be challenging. To be able to provide long-term care to the elderly, informal caregivers need to be supported.

**Methods:**

A systematic literature search was performed in Medline, PsychINFO, Ovid Nursing Database, Cinahl, Embase, Cochrane Central Register of Controlled Trials and British Nursing Index in September 2010.

**Results:**

Four systematic reviews and 10 primary studies assessing the effectiveness of caregiver support interventions were included. Overall, the effect of caregiver support interventions is small and also inconsistent between studies. Respite care can be helpful in reducing depression, burden and anger. Interventions at the individual caregivers’ level can be beneficial in reducing or stabilizing depression, burden, stress and role strain. Group support has a positive effect on caregivers’ coping ability, knowledge, social support and reducing depression. Technology-based interventions can reduce caregiver burden, depression, anxiety and stress and improve the caregiver’s coping ability.

**Conclusion:**

We identified evidence to support comprehensive multi-targeted interventions for caregivers irrespective of disease entity. These data support stakeholders when designing new avenues for the support of informal caregivers of community-dwelling frail elderly.

